# An Overview of Genes From *Cyberlindnera americana*, a Symbiont Yeast Isolated From the Gut of the Bark Beetle *Dendroctonus rhizophagus* (Curculionidae: Scolytinae), Involved in the Detoxification Process Using Genome and Transcriptome Data

**DOI:** 10.3389/fmicb.2019.02180

**Published:** 2019-09-27

**Authors:** L. Viridiana Soto-Robles, Verónica Torres-Banda, Flor N. Rivera-Orduña, Everardo Curiel-Quesada, María Eugenia Hidalgo-Lara, Gerardo Zúñiga

**Affiliations:** ^1^Escuela Nacional de Ciencias Biológicas, Instituto Politécnico Nacional, Mexico City, Mexico; ^2^Centro de Investigación y de Estudios Avanzados, Instituto Politécnico Nacional, Mexico City, Mexico

**Keywords:** genome, detoxification, MDR transporters, α-pinene, transcriptome, bark beetles, symbiont yeast

## Abstract

Bark beetles from *Dendroctonus* genus promote ecological succession and nutrient cycling in coniferous forests. However, they can trigger outbreaks leading to important economic losses in the forest industry. Conifers have evolved resistance mechanisms that can be toxic to insects but at the same time, bark beetles are capable of overcoming tree barriers and colonize these habitats. In this sense, symbiont yeasts present in the gut of bark beetles have been suggested to play a role in the detoxification process of tree defensive chemicals. In the present study, genes related to this process were identified and their response to a terpene highly toxic to bark beetles and their symbionts was analyzed in the *Cyberlindnera americana* yeast. The genome and transcriptome of *C. americana* (ChDrAdgY46) isolated from the gut of *Dendroctonus rhizophagus* were presented. Genome analysis identified 5752 protein-coding genes and diverse gene families associated with the detoxification process. The most abundant belonged to the Aldo-Keto Reductase Superfamily, ATP-binding cassette Superfamily, and the Major Facilitator Superfamily transporters. The transcriptome analysis of non-α-pinene stimulated and α-pinene stimulated yeasts showed a significant expression of genes belonging to these families. The activities demonstrated by the genes identified as Aryl-alcohol dehydrogenase and ABC transporter under (+)-α-pinene suggest that they are responsible, that *C. americana* is a dominant symbiont that resists high amounts of monoterpenes inside the gut of bark beetles.

## Introduction

Bark beetles from *Dendroctonus* genus (Curculionidae: Scolytinae) are natural agents of pines forests (Family: Pinaceae), which participate in forest regeneration and matter recycling by colonizing and killing old and weakened trees (Raffa et al., 2015). They are also regarded as disturbance agents, because some of these species undergo broadscale outbreak killing thousands of trees, thereby changing the ecological landscape and affecting the natural capital of forests (Raffa et al., 2008).

The life cycle of these insects occurs almost completely under the bark of pine trees, where they feed on phloem, reproduce and their offsprings develop (Six and Bracewell, 2015). However, to colonize the host trees and overcome their main defense system, which is a complex blend of volatile monoterpenes, non-volatile diterpenes, and sesquiterpenes, insects must circumvent the constitutive and induced resin flow as well as the highly toxic effect of some of these terpenes present in them (Franceschi et al., 2005; Seybold et al., 2006).

*Dendroctonus* species have evolved several strategies to colonize and overcome the toxic effect of the terpenes. One of them is the massive attack on their host trees, coordinated by means of aggregation pheromones (Borden, 1982), which allow recruitment of a critical number of conspecifics to quickly overcome tree resistance. Another, not less important strategy, is the development of complex interactions with different microorganisms (e.g., fungi, yeasts, and bacteria), from which insects obtain direct benefits. For example, it has been documented that microorganisms, independently of the insects’ metabolic capacities, can provide nutritional support to bark beetles (Ayres et al., 2000; Bentz and Six, 2006; Morales-Jiménez et al., 2012, 2013; Cano-Ramírez et al., 2016; Briones-Roblero et al., 2017); metabolize different xenobiotics including phenolic compounds and terpenoids that aid in the detoxification of insects (Lah et al., 2013; Cheng et al., 2016; Howe et al., 2018), and produce pheromone compounds that affect beetle behavior (Six, 2012; Davis et al., 2013). Also, the microbes increase the success of colonization of trees, cooperating in the killing of them (Six and Wingfield, 2011; Boone et al., 2013), regulate immune response of insects (Shi and Sun, 2010), as well as the interactions between them and other microorganisms (Scott et al., 2008; Adams et al., 2009; Zhou et al., 2016).

The detoxification of xenobiotics is a complex process for bark beetles and their associated symbionts. It is performed in different physiological phases where a variety of genes and proteins appear to be involved. Currently, it is well-known that in microorganisms not only enzymes like Cytochrome P450 (CYP) monooxygenases participate in xenobiotics detoxification, but also other enzymes like Glutathione S-transferases (GST), Laccases which are Multicopper-containing oxidases, Carboxylesterases, Flavin-containing monooxygenases (FMO), and Aldo-keto reductases (AKR) (Sheehan et al., 2001; Barski et al., 2008; Sehlmeyer et al., 2010; Lah et al., 2013; Marmulla and Harder, 2014; Ramya et al., 2016). In the same way, for Multidrug Resistance (MDR) transporters such as the ATP-binding cassette (ABC) Superfamily, the Major Facilitator Superfamily (MFS), and the Multidrug And Toxic compound Extrusion (MATE) transporters can also be involved in this process (Lah et al., 2013; Eisinger et al., 2018).

Despite this knowledge, few in-depth studies have explored the genes and proteins in symbionts of these bark beetles that could be involved in the detoxification process. An *in silico* study suggests that CYP monooxygenases and MFS transporters of *Candida oregonensis*, a symbiont yeast of *Dendroctonus rhizophagus*, could be involved in the metabolism of terpenoids (Hernández-Martínez et al., 2016). Besides molecular evidence has shown that an ABC transporter in *Grosmannia clavigera*, a fungus associated with *Dendroctonus ponderosae*, confers resistance to this species against monoterpenes (DiGuistini et al., 2011; Wang et al., 2013).

*Cyberlindnera americana* (Wickerham) was originally isolated from insect frass of coniferous trees (Wickerham, 1965). This yeast has been found in different life stages of *Dendroctonus* species and isolated from the body, ovarioles, and gut (where is dominant) of organisms of several geographical locations (Rivera et al., 2009; Lou et al., 2014; Briones-Roblero et al., 2017). It has been demonstrated that this species can degrade different substrates including starch and lipids (Briones-Roblero et al., 2017), and tolerate and detoxify high concentrations of toxic compounds produced by pine trees such as (+)-α-pinene (Briones-Roblero per. Comm.). Given that this yeast is a common symbiont of *Dendroctonus* species (Rivera et al., 2009; Davis, 2015), which have capacities to tolerate and degrade high concentrations of trees compounds and that the use of “omics” technologies is fundamental to document and have an overview of the genes and proteins involved in the process, the present study aims to identify the diversity of genes involved in the detoxification process in *C. americana* and to evaluate their expression after being stimulated with α-pinene, a terpene highly toxic to bark beetles and their symbionts by means of genomics and transcriptomics approaches.

## Materials and Methods

### Insect Collection, Isolation, and Taxonomic Identification of *C. americana*

*Cyberlindnera americana* ChDrAdgY46 was isolated and identified previously by Briones-Roblero et al. (2017), as described below. Emerged adults (female and male adults colonizing host trees) of *D. rhizophagus* were collected from naturally infested Arizona pines (< 3 m high, 10 cm diameter), *Pinus arizonica* Engelm. in San Juanito, Bocoyna Municipality, Chihuahua State, Mexico (27° 45′ 11″ N, 107° 38′ 06″ W). The beetles were transported to the laboratory in sterile plastic containers. The insects surface was disinfested by means of sequential rinses with sterile distilled water for 1 min, a detergent solution (10 mmol/L Tris–HCl pH 8, 1 mmol/L EDTA, 10 mmol/L NaCl, 1% SDS, 2% Triton X-100) for 1 min, a solution of 1% sodium hypochlorite for 1 min, 70% ethanol solution for 1 min, and repeated washes with sterile distilled water. To assess the efficiency of this disinfestation of yeasts and bacteria, the last washing water was inoculated in Petri dishes with yeast extract-peptone-dextrose (YPD) agar (10 g/L yeast extract, 20 g/L peptone, 20 g/L dextrose, 15 g/L agar, Difco, Detroit, MI, United States), and tryptic soy agar (TSA; 15 g/L tryptone, 5 g/L soytone, 5 g/L sodium chloride, 15 g/L agar, Difco, Detroit, MI, United States), respectively. Plates were incubated at 28°C for 48–72 h.

To remove the gut, insects were dissected in a drop of phosphate buffered solution (PBS; pH 7.4; 137 mmol/L NaCl, 2.7 mmol/L KCl, 10 mmol/L Na_2_HPO_4_, 2 mmol/L KH_2_PO_4_) under sterile conditions using fine-tipped forceps. Each gut was put into a 1.5 mL microcentrifuge tube and was homogenized in 1 mL of PBS with sterile pistils under an aseptic condition. A pool of 30 guts for the yeasts isolation was used. To obtain pure cultures, a total of 60 colonies of yeasts were randomly isolated from the plates, and the isolates were repeatedly streaked on agar plates. Axenic cultures were stored at −70°C in 40% glycerol. Lastly, the ChDrAdgY46 strain was taxonomically identified as *C. americana* based on similarity values with respect to reference sequences from GenBank and on a maximum likelihood phylogenetic analysis using ITS and 26S rRNA. The sequences of this strain are deposited in the GenBank, under accession numbers KU144269 and KU144578, respectively (Briones-Roblero et al., 2017). The strain is stored in cryogenic conditions (−70°C) at the Departamento de Microbiología from Escuela Nacional de Ciencias Biológicas del IPN and the Centro de Investigación y de Estudios Avanzados.

### Genome DNA Isolation and Sequencing

*Cyberlindnera americana* ChDrAdgY46 was grown at 28°C for 24 h on 5 mL YPD broth (1% yeast extract, 2% peptone, and 2% dextrose, Difco, Detroit, MI, United States). Genomic DNA (gDNA) was obtained from the yeast suspension with the RiboPure Yeast Kit (Ambion, Carlsbad, CA, United States) following the manufacturer’s protocol, with some slight modifications: DNA elution was performed with 1X TE buffer and treated with RNase A 10 mg/mL (Thermo Fisher Scientific, Waltham, MA, United States) at 37°C for 1 h. The total gDNA quantity and quality were measured in a NanoDrop 2000 (Thermo Fisher Scientific), whole-genome sequencing was done by Otogenetics Corporation (Norcross, GA, United States). Fragmentation of gDNA was achieved using a Bioruptor Sonicator (Diagenode Inc., Denville, NJ, United States). Fragmented gDNAs size distribution and concentration were tested using an Agilent Bioanalyzer 2100 (Agilent Technologies Inc., Santa Clara, CA, United States) and NanoDrop 2000, respectively. DNA libraries were constructed by using an SPRI works HT Reagent Kit (Beckman Coulter Inc., Indianapolis, IN, United States), and whole-genome sequencing was performed using Illumina (Illumina Inc., San Diego, CA, United States) Hi-Seq 2500 platform (125 × 2 cycles).

### Genome Assembly and Functional Annotation

Data sets were analyzed for quality using FASTQC^1^ v 0.11.7. Reads were prefiltered to remove sequencing adapters and low quality reads using Trimmomatic v 0.38 (Bolger et al., 2014), using a sliding window of k-mer = 5 to removed leading or trailing bases with average Phred quality score lower than 28, as well as reads below 25 pb. *De novo* genome assembly was performed using Velvet v 1.2.10 (Zerbino and Birney, 2008) with different k-mer settings from 19 to 99 nt. The assembly with the longest scaffold N50, a k-mer = 39, was selected.

Gene prediction was performed using AUGUSTUS v 2.5.5 (Stanke and Morgenstern, 2005) with *Saccharomyces cerevisiae* as reference species. For quantitative assessment of assembly and annotation completeness, we used Benchmarking Universal Single-Copy Orthologs (BUSCO v 3) comparing with the library of Saccharomycetales orthologous genes. Gene Ontology (GO) annotation was carried out in the SwissProt/UniProt database (download March/2018) using BlastP and BlastX (*e*-value cut-off ≤ 10^–5)^, in the Pfam database (download March/2018) using the HMMER v 3.1.2 software (Finn et al., 2011), and the distribution of GO terms was plotted with WEGO v 2.0 (Ye et al., 2006). The WebMGA server (Wu et al., 2011) was used against the KOG (Eukaryotic Orthologous Groups) database (Accessed July, 2019).

### Identification of Detoxification Enzymes and MDR Transporters

Putative enzymes associated with the detoxification process present in *C. americana*, such as CYP monooxygenases, GST, Carboxylesterases, FMO, Multicopper oxidases, and the AKR superfamily, as well as MDR transporters were identified through the protein domains (Pfam database), and verified with the results of BlastP with expectation values (*e*-value) of 0.0001. Particularly, the BlastP analyses of MDR transports were performed with mask off segments of the query sequence that had lower compositional complexity. Segments consisting of short internal repeats were filtered with SEG and XNU, using a word-size of three letters and BLOSUM62 matrix. Sequences with higher scores (HSP) and *e*-value of 0.0001 were retained, and MDR transporters with ≥ 5 transmembrane helices (TMH) were recovered with HMMTOP v 2.1 (Tusnády and Simon, 2001), PHOBIUS v 1.05 (Käll et al., 2004) and TMHMM v 2.0 (Krogh et al., 2001). Finally, the functional analyses of domain architectures from all these sequences were performed using the InterProScan software (Quevillon et al., 2005).

Enzymes of the AKR superfamily were classified into specific families according to the BlastP results and phylogenetic analysis by maximum likelihood. Multiple sequence alignment was carried out using MUSCLE v 3.8.31 (Edgar, 2004), and the phylogenetic tree was performed directly in PhyML v 3.0 online. The best evolutionary model for protein data was selected using the Akaike method and the consistency at each node was assessed by aLRT SH-like and bootstrapping (100 bootstrap) (Guindon et al., 2010).

Following two strategies, transporters belonging to the ABC and MFS Superfamilies were classified into specific families. First, we performed a BlastP of transporters into the Transporter Classification Database^2^, the data set was filtered using SEG and XNU retaining HSP with *e*-value of 0.0001; second, the phylogenetic analyses were conducted as previously described.

Lastly, to know if the diversity of genes associated with the process of detoxification in *C. americana* is significant with respect to other yeasts, we compared the genes associated to this process in this yeast with those of *Cyberlindnera jadinii* (GCF_001661405.1), a species of the same phylogenetic group (Shen et al., 2018), as well as *Clavispora lusitaniae* (GCF_000003835.1) and *Candida tropicalis* (GCF_000006335.3), two species well-studied for their capacity of antifungal drug resistance. For this, we identified and compared the Pfam of all genes, which were confirmed employing a BlastP and through the searching of the corresponding domains by InterProScan, and the MDR transporters with at least 5 TMH were recovered as previously described.

### Localization Prediction

Physicochemical characteristics such as the molecular mass and isoelectric point (pI) of detoxification enzymes and MDR transporters were predicted using a Protein isoelectric point calculator server (Kozlowski, 2016). The subcellular localization prediction was performed with Protcomp-AN v 9.0 (Softberry, Inc.) and the signal peptide was predicted with the SignalP v 5.0 (Armenteros et al., 2019).

### α-Pinene Treatment, RNA Extraction, and Sequencing

*Cyberlindnera americana* ChDrAdgY46 was grown in 100 mL of 10% YPD broth, 0.1% (v/v) (+)-α-pinene was added (Sigma-Aldrich, St. Louis, MO, United States) (α-pinene stimulated, PS), and incubated at 28°C for 48 h at 150 rpm. The concentration of 0.1% (+)-α-pinene is higher than those found in the gut of some *Dendroctonus* species (López et al., 2011; Chiu et al., 2017). A yeast culture without monoterpene (non-α-pinene stimulated, NPS) was included as control and incubated at the same conditions. Three biological replicates were made for each assay. Total RNA was extracted from each yeast culture with the RiboPure Yeast Kit (Ambion) according to the manufacturer’s protocol. Using Agilent Bioanalyzer 2100 (Agilent Technologies), the integrity of RNA was evaluated.

Based on the manufacturer’s protocol, RNA libraries were constructed with the TruSeq Stranded mRNA Library Prep Kit (Illumina Inc., San Diego, CA, United States). Libraries were sequenced using the Illumina NextSeq 500 platform (75 × 2 cycles) at the Unidad Universitaria de Secuenciación Masiva y Bioinformática of the Instituto de Biotecnología-UNAM (Cuernavaca, Mor, Mexico).

### Transcriptome Assembly and Functional Annotation

To remove sequencing adapters and low-quality reads, sequences were pre-filtered in Trimmomatic v 0.38 (Bolger et al., 2014), using the parameters previously described in the genome depuration. A *de novo* transcriptome assembly was performed into a single combined assembly, using the short-read assembly program Trinity v 2.6 with default parameters (Haas et al., 2013).

The transcriptome annotation was performed using the Trinotate v 3.1.1 pipeline^3^. For quantitative assessment of assembly and annotation completeness, BUSCO v 3 was applied comparing with the library of Saccharomycetales orthologous genes. The homology of all assembled putative genes was searched with BlastX and BlastP (*e*-value cut-off ≤ 10^–5^) against the SwissProt/UniProt database (download March/2018). Open reading frames were predicted using TransDecoder^4^ v 5.3.0, and conserved protein domains were identified with HMMER v 3.1.2 (Finn et al., 2011) against the Pfam domain database (download March/2018). To determine putative gene functions, all transcripts were analyzed in Gene Ontology (GO) terms and classified with WEGO v 2.0 (Ye et al., 2006). Finally, in order to identify the orthologous and paralogous proteins of eukaryotes, the WebMGA server (Wu et al., 2011) was used against the KOG (Eukaryotic Orthologous Groups) database (Accessed April, 2018).

### Gene Expression Profiling and Differential Analysis

After data cleaning described above, reads were aligned to the genes predicted from the genome using Bowtie2. Then, RSEM tool (Li and Dewey, 2011) was used to identify the gene expression levels in all samples. TPM (transcripts per million reads) metric was used for normalization and lowly expressed genes (< 5 TPM) were filtered. Recommended parameters by Trinity were used for Bowtie2 and RSEM.

To identified the differentially expressed genes (DEG), the reads genes counts matrix was used in the Integrative Differential Expression Analysis for Multiple Experiments server (Jiménez-Jacinto et al., 2019) using Bioconductor edgeR, NOISeq, and DESeq2 packages within this server with the following parameters: fold change (log_2_FC) ≥ 1, *p*-value ≤ 0.05, FDR ≤ 0.05, and CPM = 1.

### GO and KEGG Enrichment Analysis of DEG

A Gene Ontology (GO) enrichment analysis of differentially expressed genes was implemented by the Bioconductor package Goseq with default parameters. This analysis eliminates the influence of gene length in the determination of the enriched GO terms with corrected *p*-value < 0.05. Lastly, we also analyzed the enrichment of differentially expressed genes in KEGG pathways with KOBAS software v 3.0 using default parameters (Xie et al., 2011).

## Results

### Genome Analysis: *De novo* Assembly and Functional Annotation

A total of 19 788 540 reads were obtained from sequencing the DNA library of *C. americana*, after data cleaning, we obtained 17 352 666 reads. Obtaining the assembled genome, which consisted of 11 473 843 bp across 176 scaffolds (≥ 200 bp) with a G + C content of 41.42%. The longest scaffold had a length of 677 423 bp, and the N50 was 188 956 bp. The BUSCO results showed that out of 1711 single-copy orthologs for Saccharomycetales, our assembly is 92.87% complete (1582 complete single-copy orthologs and 7 complete duplicated orthologs), while 2.57% (44) is fragmented and 4.56% (78) is missing (Table 1).

**TABLE 1:** Genome assembly statistics of *C. americana.*

**Details**	**Value**
Total length of sequence (bp)	11 473 843
No. of Scaffolds	176
Minimum length of contigs/scaffolds (bp)	200
Maximum length of contigs/scaffolds (bp)	677 423
N50 (bp)	188 956
GC content (%)	41.42
No. of genes predicted	5752
BUSCO completeness (%)	92.87

**FIGURE 1:**
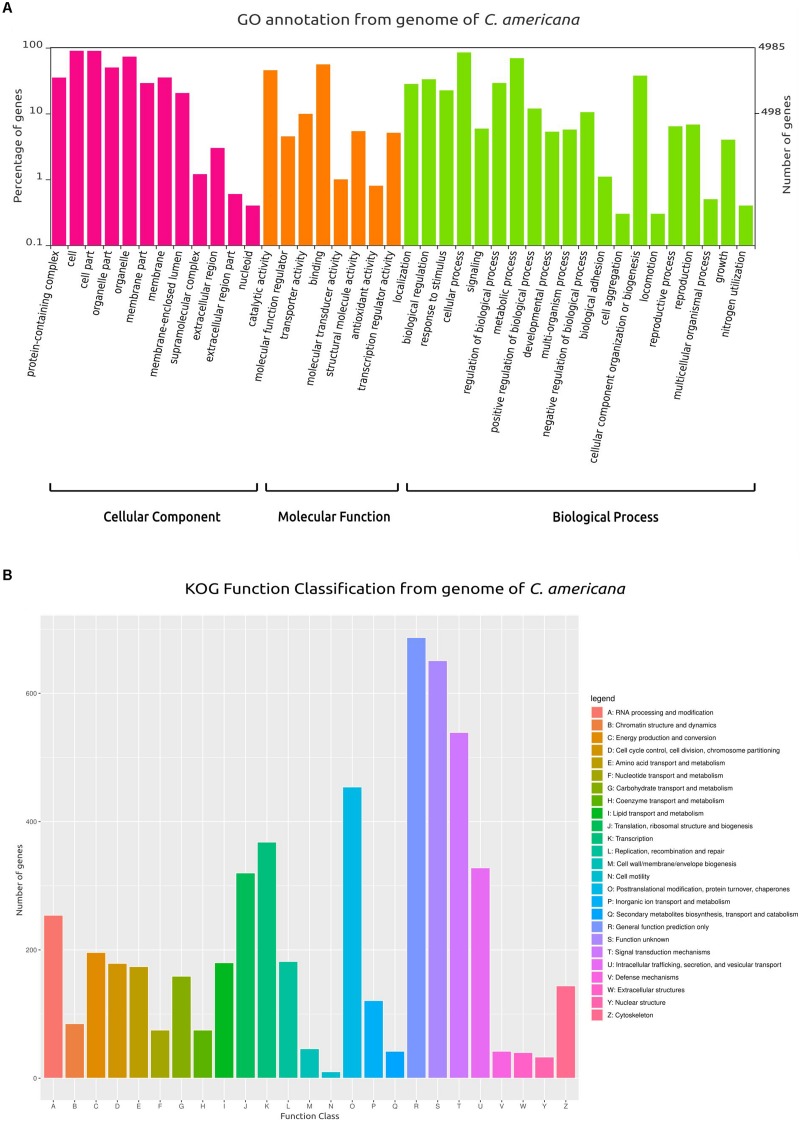
Gene Ontology (GO) and Clusters of Orthologous Groups (KOG) plots. **(A)** Classification and functional distribution of the inferred proteome from the genome of *C. americana*, according to the three major hierarchical Gene Ontology terms: Biological Process, Molecular Function, and Cellular Component. Obsolete terms and values ≤ 0.2% were not represented. **(B)** Distribution of KOG annotation of genes related to A–Z classes.

From the total of 5752 protein-coding genes (Supplementary Data Sheet S1), 4886 (84.53%) were identified using BlastX and 4867 (84.86%) using BlastP matched with sequences deposited in SwissProt/UniProt, while 4748 (82.46%) genes were identified using HMMER in Pfam. Also, 4985 were categorized in at least one of the following ontologies of the GO database: cellular components, molecular function, and biological process (Figure 1A). Similarly, based on KOG annotation, a total of 5384 (93.60%) genes were classified in the different groups (A–Z, Figure 1B).

### Sequences Analysis of Detoxification Enzymes and MDR Transporters

The results of Pfam, InterProScan, and SwissProt/UniProt identified 212 sequences detoxifying-related genes as CYP monooxygenases, FMO, GST, Multicopper oxidases, enzymes of the AKR superfamily, and Carboxylesterases, as well as MFS, ABC, and MATE (Table 2).

**TABLE 2:** Genes involved in the detoxification process of *C. americana.*

**Enzyme**	**Pfam**	**No. of sequences confirmed by Blast and InterProScan**
Aldo-keto reductases	PF00248	21
Carboxylesterase	PF00135	5
Cytochrome p450 monooxygenase	PF00067	5
Flavin-containing monooxygenase	PF00743	6
Glutathione S-transferase	PF13409, PF14497, PF02798, PF13417, PF00043, PF13410	3
Multicopper-containing oxidases	PF07732, PF00394, PF07731	1
**Transporter**		
ABC	PF00664, PF00005, PF06472, PF00004	20
MATE	PF01554	6
MFS	PF07690, PF16983, PF05631, PF00083, PF12832, PF06779, PF13347, PF05977	145

CYP monooxygenases varied from 481 to 536 amino acids, their predicted molecular weights ranged from 55.75 to 61.62 kDa, and their pIs from 5.44 to 8.37. The GST enzymes showed similar lengths of 237-324 amino acids. The Multicopper oxidase found was predicted as extracellular laccase of 659 amino acids. FMO enzymes were found with lengths varying from 461 to 638 amino acids with weights ranged from 52.08 to 72.11 kDa. Five Carboxylesterases of approximately 550 amino acids were recognized, three of them were predicted as extracellular enzymes but just two of them showed a predicted signal peptide (for details on these enzymes see Supplementary Table S1).

**FIGURE 2:**
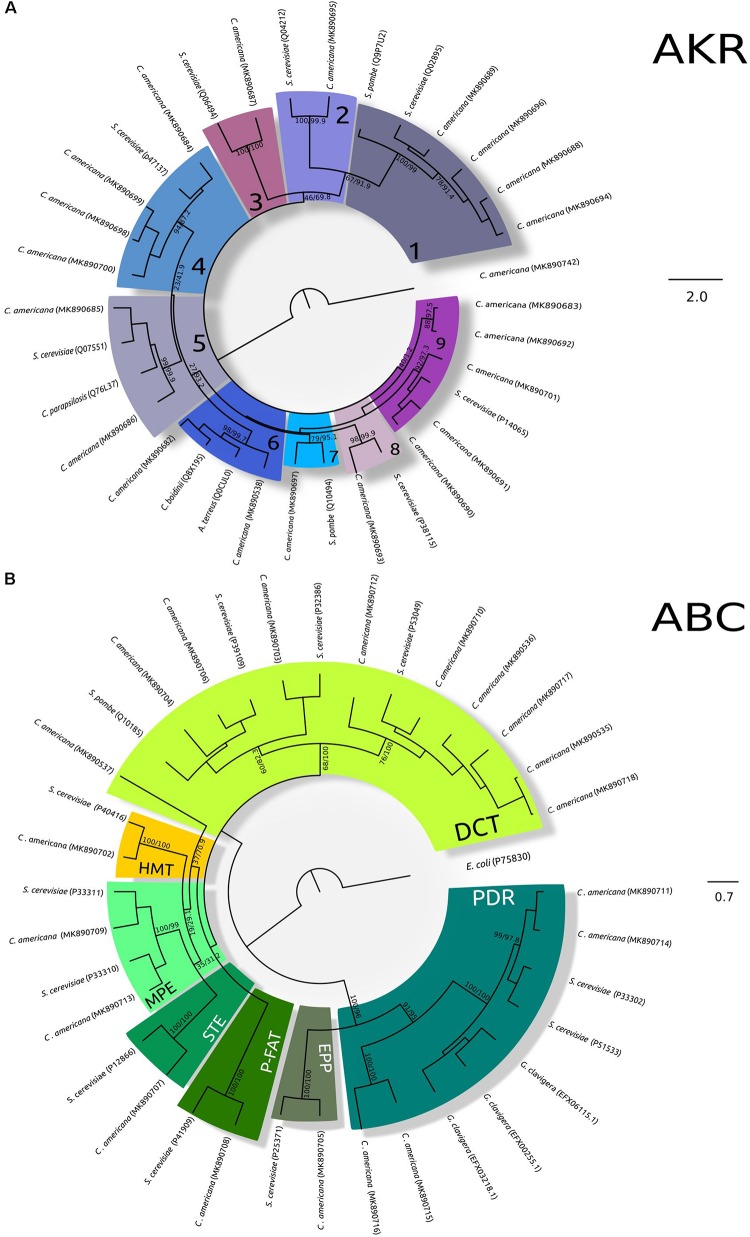
Maximum-likelihood trees of AKR and ABC transporters. **(A)** Phylogeny of AKR. The analysis was performed using the amino acid substitution model LG + G + F with a gamma parameter of 1.582, bootstrap/aLRT SH-like values shown at nodes. The alcohol dehydrogenase (non-member of the AKR Superfamily) (from *C. americana* was used as outgroup. 1, Aryl-alcohol dehydrogenase; 2, D-arabinose 1-dehydrogenase; 3, Pyridoxal reductase; 4, Oxidoreductase; 5, NAD(P)H-dependent reductase; 6, NAD(P)H-dependent D-xylose reductase; 7, Aldehyde reductase I; 8, D-arabinose dehydrogenase (NAD(P)+) heavy chain; 9, Glycerol 2-dehydrogenase (NAD(P)+). Accession numbers of TCDB/GenBank sequences are shown in brackets. **(B)** Phylogeny of ABC transporters. The analysis was performed using the amino acid substitution model VT + G + I + F with a gamma parameter of 2.312 and a proportion of invariable sites of 0.005, bootsrap/aLRT SH-like values shown at nodes. The ABC III from *E. coli* (P75830) was used as outgroup. DCT, drug conjugate transporter; P-FAT, peroxisomal fatty acyl CoA transporter; HMT, heavy metal transporter; MPE, mitochondrial peptide exporter; STE, a-factor sex pheromone exporter; PDR, pleiotropic drug resistance; EPP, eye pigment precursor transporter. Accession numbers of TCDB/GenBank sequences are shown in brackets.)

On the other hand, twenty-one AKR enzymes from 283 to 356 amino acids were found, their predicted molecular weights varied from 32.51 to 40.80 kDa, and their pIs from 4.82 to 6.22; the localization predictions were in cytoplasm, plasma membrane, and bounding membrane of Golgi and mitochondria (Supplementary Table S1). Belonging to these enzyme groups: four Aryl-alcohol dehydrogenases (AAD) enzymes, four Oxidoreductases, two NAD(P)H-dependent reductases, two D-arabinose 1-dehydrogenases, five Glycerol 2-dehydrogenases (NAD(P)+), two NAD(P)H-dependent D-xylose reductases, one Aldehyde reductase I, one Pyridoxal reductase, one D-arabinose 1-dehydrogenase, and one D-arabinose dehydrogenase (NAD(P)+) heavy chain were identified (Supplementary Data Sheet S1). The phylogenetic analysis showed consistent groups for almost all enzymes, only two from five Glycerol 2-dehydrogenases (NAD(P)+) were grouped in a different group (Figure 2A).

Twenty ABC transporters showed lengths from 540 to 1653 amino acids, molecular weights from 59.92 to 185.52 kDa, and pIs from 5.01 to 8.64; ABC transporters were predicted in the plasma membrane, and membranes of mitochondria, vacuole, peroxisome, and endoplasmic reticulum. In the case of incomplete sequences, the prediction could be uncertain, specifically in the case *CaABCC10* where its closest homologous is localized in the plasma membrane (Supplementary Table S1). The topology of the ABC transporter tree showed consistency with the two ATP-binding cassette subfamilies: ABC I and ABC II; excepting *CaABCC5* that was associated with ABC-B transporters. The ABC I subfamily was integrated by the drug conjugate transporter (DCT) from ABC-C transporters; the peroxisomal fatty acyl CoA transporter (P-FAT) from ABC-D transporters; the heavy metal transporter (HMT); the mitochondrial peptide exporter (MPE); and the a-factor sex pheromone exporter (STE) from ABC-B transporters. The ABC II subfamily was integrated by the pleiotropic drug resistance (PDR) including GcABC-G1-3 from filamentous fungus *G. clavigera*, and the eye pigment precursor transporter (EPP) from ABC-G transporters (Figure 2B).

**FIGURE 3:**
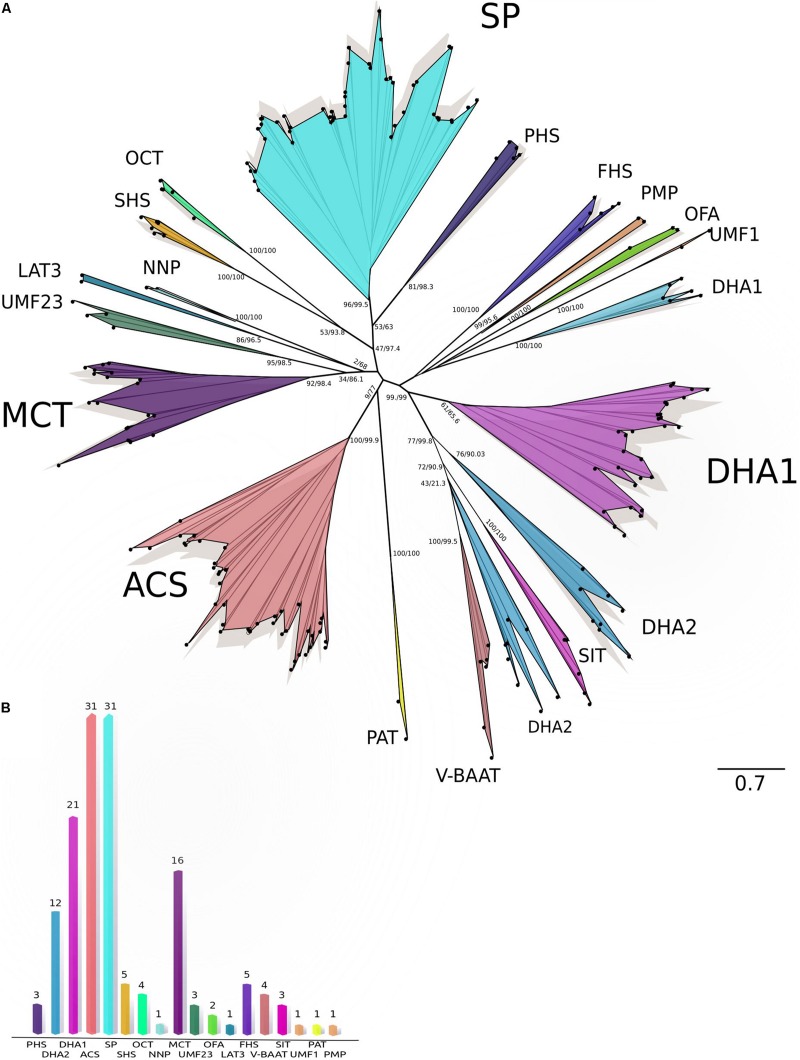
Maximum-likelihood tree and Classification of MFS transporters. **(A)** Phylogeny of MFS sequences. The maximum-likelihood tree of MFS based on amino acid sequences from *C. americana* plus TCDB sequences. The analysis was performed using the amino acid substitution model VT + G + F with a gamma (parameter of 3.876. Bootstrap/aLRT SH-like values are shown at nodes. **(B)** Classification of MFS families. The graph shows 145 putative MFS classified into 18 different MFS families. ACS, anion:cation symporter family; DHA1 and DHA2, drug:H+ antiporter 1–2 families; FHS, fucose:H+ symporter family; LAT3, L-amino acid transporter-3 family; MCT, monocarboxylate transporter family; NNP, nitrate/nitrite porter family; OCT, organic cation transporter family; OFA, oxalate:formate antiporter family; PAT, peptide-acetyl-coenzyme a transporter family; PHS, phosphate:H+ symporter family; PMP, putative magnetosome permease family; SHS, sialate:H+ symporter family; SIT, siderophore-iron transporter family; SP, sugar porter family; UMF1, unidentified major facilitator-1 family; UMF23, unidentified major facilitator-23 family; V-BAAT, vacuolar basic amino acid transporter family.)

The MFS transporters (145 transporters) varied from 440 to 699 amino acids, their predicted molecular weights varied from 48.07 to 76.93 kDa, and pIs from 4.89 to 8.49. The MFS transporters were predicted in plasmatic membrane and bounding membrane of endoplasmic reticulum, vacuole, and Golgi (Supplementary Table S1). The maximum likelihood phylogenetic analysis of MFS transporters (Figure 3A) showed the consistent integration of diverse and well-supported groups, according to their functional classification, except for some DHA1 and DHA2 members. The MFS transporters were classified into 18 different families with the most abundant being those associated with sugar porter (SP), anion:cation symporter (ACS), drug:H+ antiporter 1-2 (DHA1, DHA2), and monocarboxylate transporter (MCT), while less abundant were the phosphate:H+ symporter (PHS), sialate:H+ symporter (SHS), organic cation transporter (OCT), unidentified major facilitator-23 (UMF23), fucose:H+ symporter (FHS), vacuolar basic amino acid transporter (V-BBAT), and siderophore-iron transporter families (SIT) (Figure 3B).

Lastly, the lengths of six MATE transporters ranged from 496 to 645 amino acids, their molecular weights ranged from 54.09 to 71.16 kDa, and pIs from 2.33 to 7.16; the predicted location of these transporters was vacuolar (Supplementary Table S1).

### Transcriptome Analysis: *De novo* Assembly and Functional Annotation

A total of 53 782 804 reads were obtained from sequencing the RNA libraries of *C. americana*. After data cleaning, we obtained 46 295 130 reads, which were assembled into 8531 transcripts with N50 length 2339 bp with a G + C content of 42.36%. The length of the transcripts varied from 200 to 12 817 bp with an average length of 1501.09 bp (Supplementary Figure S1), and 99.87% of clean reads were mapped to the *de novo* assembly. BUSCO results showed that out of 1711 single-copy orthologs for Saccharomycetales, our assembly is 89.60% complete (1216 complete single-copy orthologs and 317 complete duplicated orthologs), while 7.36% (126) is fragmented and 3.04% (52) is missing.

From the total of transcripts 5696 (66.77%) and 5736 (67.24%) unigenes significantly matched with sequences deposited in SwissProt/UniProt by BlastX and BlastP, respectively; while 5430 (63.65%) unigenes matched in Pfam.

**FIGURE 4:**
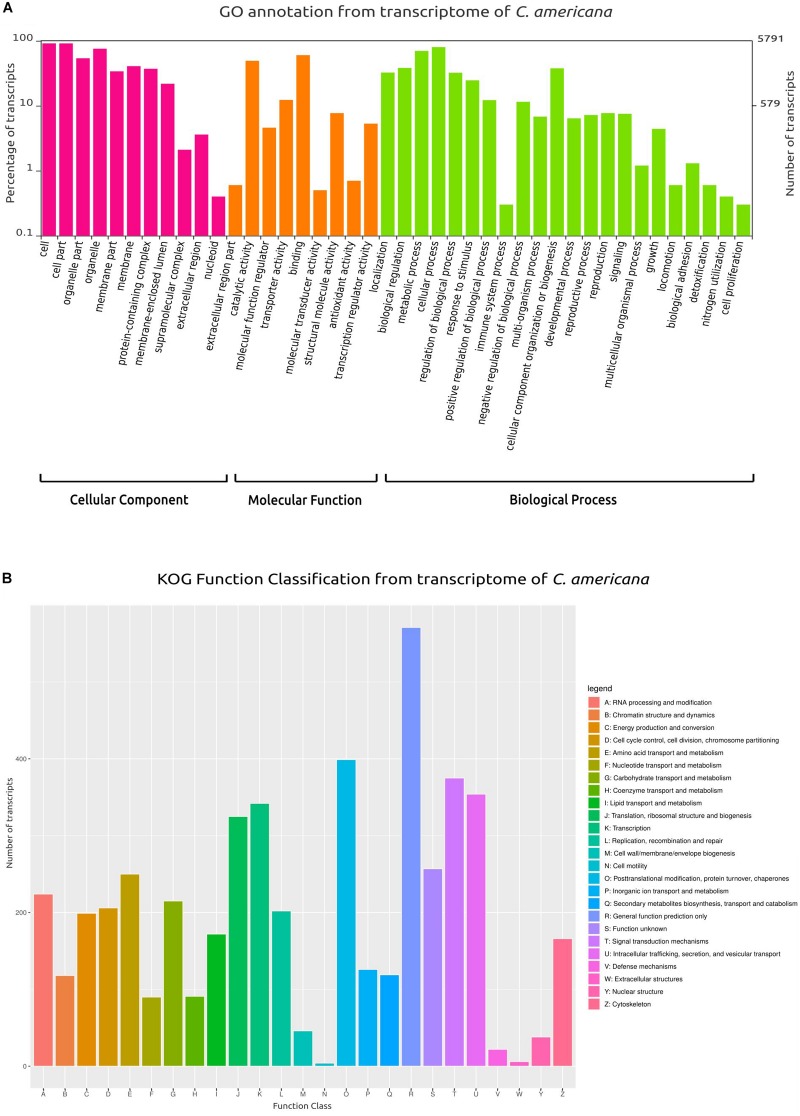
Gene Ontology (GO) and Clusters of Orthologous Groups (KOG) plots. **(A)** Classification and functional distribution of the inferred proteome from the transcriptome of *C. americana*, according to the three major hierarchical Gene Ontology terms: Biological Process, Molecular Function, and Cellular Component. Obsolete terms and values ≤ 0.2% were not represented. **(B)** Distribution of KOG annotation of transcripts related to A–Z classes.

From the total transcripts, 5791 (67.88%) showed a GO annotation, categorized in three main groups: cellular components, molecular function and biological process (Figure 4A). Similarly, based on KOG annotation, a total of 4751 (60.86%) transcripts were classified in the different groups (A–Z, Figure 4B). Among these groups, the main group was the general function (class R) with 571 transcripts, followed by post-translational modification/protein turnover/chaperones (class O) with 399 transcripts, signal transduction mechanisms (class T) with 375 transcripts, and intracellular trafficking/secretion/vesicular transport (class U) with 354 transcripts. Furthermore, 1063 transcripts were associated with transport and metabolism: amino acid transport and metabolism with 250 (class E), carbohydrate transport and metabolism with 215 transcripts (class G), lipid transport and metabolism with 172 transcripts (class I), inorganic ion transport and metabolism with 126 transcripts (class P), secondary metabolites biosynthesis, transport and catabolism with 119 transcripts (class Q), coenzyme transport and metabolism with 91 transcripts (class H), and lastly nucleotide transport and metabolism with 90 transcripts (class F).

Lastly, whereas the comparison genome and transcriptome showed > 89% complete orthologs, the number of complete duplicated transcripts was higher in the transcriptome than the number of complete duplicated orthologs in the *C. americana* genome. A GO and a KOG comparison between the annotation of genome and transcriptome was done, founding GO terms exclusives in these assemblies (Supplementary Table S2).

With respect to genes presumably involved in the detoxification process, the comparison showed that 32 transcripts had their counterpart in the genome, and only nine genes did not have transcripts. Likewise, 166 transcripts associated with MDR transporters had representatives in the genome, and only five genes did not present transcripts (Table 3).

**TABLE 3:** Summary of genes involved in the detoxification process of *C. americana.*

	**Genes**	**Transcripts**	**Genes expressed**
**Enzyme**	**(Genome)**	**(Transcriptome)**	**with > 5 TPM**
Aldo-keto reductases	21	18	18
Carboxylesterase	5	4	3
Cytochrome p450 monooxygenase	5	4	4
Flavin-containing monooxygenase	6	5	4
Glutathione S-transferase	3	0	2
Multicopper-containing oxidases	1	1	0
**Transporter**			
ABC	20	19	13
MATE	6	6	5
MFS	145	141	93

### Gene Expression Profiling and Differential Analysis

The gene expression profiling, using the genome of *C. americana* (5752 genes) and the RNA-seq (46 295 130 reads), allowed to identify that 5175 genes are being expressed, where the 96.58% of clean reads (RNA-Seq) were mapped, additionally from the genes involved in the detoxification process previously identified in the genome of *C. americana* (212 genes) 142 genes were expressed (≥ 5 TPM at least in three libraries) (Table 3 and Supplementary Table S3).

**FIGURE 5:**
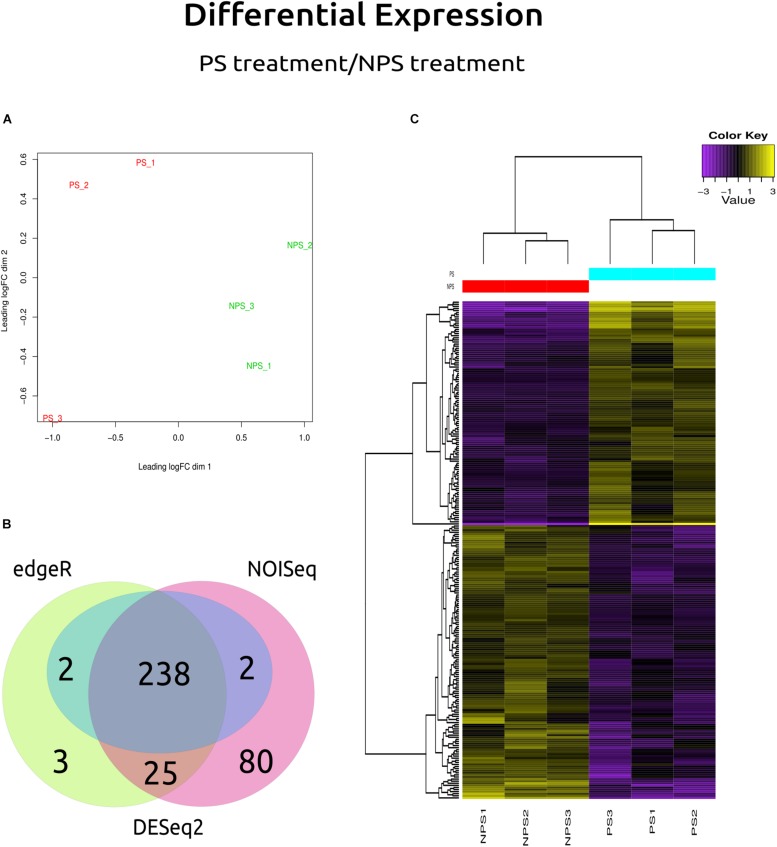
Differential gene expression results. **(A)** MDS plot: Multidimensional Scaling plot shows variation among RNA-Seq samples, distance between sample labels indicates dissimilarity. **(B)** The Venn diagram shows the genes that were reported by each method in a particular way (DESeq2, NOISeq, EdgeR, or DESeq), and which were reported by all (those shown at the center). **(C)** Heatmap: yellow color denotes a high level of expression, while purple indicates low expression, NPS1, NPS2, and NPS3 denote the biological replicates for samples without (+)-α-pinene, while PS1, PS2, and PS3 denote the three biological replicates with (+)-α-pinene.

Transcriptional patterns of the genes expressed by *C. americana* were clustered by treatment (Figure 5A). The differential analysis showed that of all the genes differentially expressed (Figure 5B), 238 genes were identified by DESeq2, NOISeq, and EdgeR software. Of these, 131 genes (55.04%) were downregulated and 107 (44.96%) upregulated under (+)-α-pinene stimulus (Supplementary Table S4). Hierarchical clustering analysis of 238 DEG of *C. americana* allowed the identification of ten genes with the highest difference in their expression levels between the two assay conditions (Table 4). The rest of them apparently is involved in the central metabolism of lipids, carbohydrates, amino acids, nucleotides, among others (for details on these genes see Supplementary Table S4). A heatmap was constructed with EdgeR results with the 238 genes differentially expressed (Figure 5C). Within these, one ABC transporter gene, one AKR, and three MFS transporters showed upregulation, whereas one CYP monooxygenase showed downregulation, as well as 23 MFS transporters, highlighting the anion:cation symporters, the monocarboxylate transporters, and the sugar porters.

**TABLE 4:** Top 10 differentially expressed genes of *C. americana* under (+)-α-pinene stimulus.

**Name**	**logFC**	**logCPM**	***P*-value**	**FDR**	**Regulation**
Pleiotropic ABC efflux transporter of multiple drugs (*CaABCG1*)	–6.187	10.047	2.32E-104	1.30E-100	UP (+)-α-pinene
Urea active transporter	4.699	6.786	7.37E-056	1.37E-052	DOWN (+)-α-pinene
Aryl-alcohol dehydrogenase (*CaAAD1*)	–4.485	7.096	4.48E-070	1.25E-066	UP (+)-α-pinene
Hypothetical protein	–4.374	2.754	3.13E-021	1.17E-018	UP (+)-α-pinene
Protein MPA43	–4.118	5.790	3.25E-030	3.03E-027	UP (+)-α-pinene
Aryl-alcohol dehydrogenase	–4.000	8.251	3.58E-050	5.00E-047	UP (+)-α-pinene
Hypothetical protein BON22_5103	3.830	2.688	5.20E-013	7.42E-011	DOWN (+)-α-pinene
Uncharacterized protein YJR098C	–3.715	6.783	7.55E-032	8.45E-029	UP (+)-α-pinene
Acid phosphatase	3.501	3.282	4.15E-009	3.18E-007	DOWN (+)-α-pinene
MFS transporter, The Sialate:H + Symporter (*CaMfs42*)	3.434	5.613	9.43E-023	5.28E-020	DOWN (+)-α-pinene

### Functional Analysis

The functional analysis identified enriched GO terms and KEGG pathways involved by the DEG (Supplementary Table S4). The GO enrichment analysis showed upregulated genes assigned to the categories: cellular component, molecular function, and biological process. Within the first category, the highest number of genes was observed in membrane part (37) and intrinsic component of membrane (30); in the second category, the main functions were transferase activity (21) and transferase activity transferring phosphorus-containing groups (11); lastly, in the last category, the main processes were the lipid metabolic process (26) and cellular lipid metabolic process (21). The same categories were observed for downregulated genes, where the cellular component category showed the highest number of genes associated to membrane part (51) and intrinsic component of membrane (51) subcategories; in the molecular function category, the main functions were transporter activity (39) and transmembrane transporter activity (37); lastly, for biological process, the main subcategories were transport (48), establishment of localization (48), localization (48), and transmembrane transport (38).

Furthermore, among the significant GO terms (Supplementary Table S4), we found that 11 upregulated genes induced by (+)-α-pinene were involved in “response to stimulus”: response to furfural (GO:1901426), positive regulation of transcription from RNA polymerase II promoter involved in unfolded protein response (GO:0006990), signal transduction (GO:0007165), endoplasmic reticulum unfolded protein response (GO:0030968), response to cation stress (GO:0043157), cellular response to cation stress (GO:0071473), and drug export (GO:0046618). Besides, three GO terms included in “response to stimulus” of downregulated genes were associated with riboflavin transport (GO:0032218), regulation of DNA double-strand break processing (GO:1903775), and quinolinic acid transmembrane transport (GO:1903222).

The KEGG enrichment analysis showed that upregulated genes were associated to metabolic pathways (ID: 01100) with 23 genes, biosynthesis of secondary metabolites (ID: 01110) with 10 genes, and glycerophospholipid metabolism (ID: 00564) with nine genes. The downregulated genes were assigned to terms metabolic pathways (14 genes), biosynthesis of antibiotics (9 genes, ID: 01130), and biosynthesis of secondary metabolites (9 genes).

## Discussion

Bark beetles have faced the toxic compounds of their host plants through different enzymes and molecular mechanisms (Robert et al., 2013; Dai et al., 2016, 2018; Chiu et al., 2019) of their own or from different symbiont microbes such as yeast, fungi, and bacteria. Some studies have shown that bark beetles-associated bacterial and filamentous fungi have developed different strategies and molecular mechanisms to excrete, metabolize into non-toxic compounds or sequester diverse terpenoid compounds (DiGuistini et al., 2011; Adams et al., 2013; Haridas et al., 2013; Lah et al., 2013). In the case of yeasts, few *in vitro* or *in vivo* studies have evaluated their metabolic abilities and participation in this process (Marmulla and Harder, 2014). The information available suggests that fungi may have molecular mechanisms that contribute significantly to their detoxification process as well as that of their hosts, favoring survival and successful adaptation to them and other plant symbionts.

Our results showed that *C. americana* has a genome size of approximately 11.5 Mb with 5752 putative protein-coding genes, and a G + C content of 41.42%, which agrees with a recent report for this species whose genome size is 11.6 MB and G + C content of 42.10% (PRJNA429441, genome not annotated) (Shen et al., 2018). The features of genome of this study are also similar to those of close yeast species such as *Cyberlindnera fabianii* [PRJEB6138] involved in fermentation and water treatment (Freel et al., 2014) and *C. jadinii* [PRJNA374042] a strain from clinical sources (Riley et al., 2016), with genome sizes of approximately 12.3 Mb and 13 Mb, gene numbers of 6084 and 6184 and a G + C content of 44.35 and 44.60%, respectively. Additionally, a comparison of BUSCO results of *C. americana* ChDrAdgY46 and other *Cyberlindnera* species shows similar results with a completeness > 90% (Supplementary Figure S2).

The genome of *C. americana* shows the presence of diverse genes coding for enzymes such as AKR, carboxylesterases, CYP monooxygenases, FMO, GST, and a multicopper oxidase, but the number of these enzymes is lower than the MDR transporters. The comparison among the genomes of *C. americana, C. jadinii, C. tropicalis*, and *C. lusitaniae* showed the presence of multicopper oxidases enzymes only in *C. americana* and *C. lusitaniae*, and a number similar of genes coding for FMO and GST enzymes, as well as MATE transporters among four species. In this comparison also highlight the presence only in *C. americana* of genes encoding carboxylesterases, and a comparable number of AKR genes among the four yeast species (Supplementary Table S5). Also, it is notable the relatively low number of CYP monooxygenases in the *C. americana* compared to other yeasts and filamentous fungi (Supplementary Table S5, including in it Chen et al., 2014 data). Lastly, *C. americana* has a relatively low number of ABC transporters and a high number of MFS transporters compared to other filamentous fungi (Supplementary Table S5, including in it Coleman and Mylonakis, 2009 data).

**FIGURE 6:**
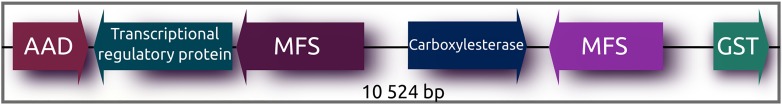
Genomic localization of the upregulated Aryl-alcohol dehydrogenase *CaAAD1* gene. *CaAAD1* gene forms a cluster together with a transcriptional regulatory protein, two MFS transporters, a Carboxylesterase, and a Glutathione S-transferase.

In this study, few differences at the transcriptional levels of most of the above detoxifying enzymes were detected between the two conditions assayed (stimulated and non-stimulated yeast). Among them, the AAD (*CaAAD1*) gene showed a significantly stronger expression (fold change ≥ 4) in the presence of (+)-α-pinene. Interestingly, despite the functional role of AAD is not well known, it has been reported that some AADs could be involved in the metabolism of aromatic compounds, detoxification of aromatic aldehydes and the response to oxidative stress (Muheim et al., 1991). In addition, studies performed with *Pleurotus ostreatus* (pearl oyster mushroom) have demonstrated that they are involved in the detoxification of compounds like 5-hydroxymethylfurfural (HMF) (Feldman et al., 2015). Moreover, the transcriptional responses of these genes to furfural and HMF in *S. cerevisiae* have also been recorded (Almario et al., 2013). Also, it has recently been documented that *AAD* genes exhibit activity in lignin degradation and are widely distributed in wood-saprophyte fungi and yeasts that have a ligninolytic habitat (Yang et al., 2017), as might be the case for *C. americana*, isolated from the bark and phloem of *P. arizonica* and *Pinus engelmannii* (Gonzalez-Escobedo et al., 2019). The observed transcriptional response of the *CaAAD1* gene induced by (+)-α-pinene indicates that additional studies are necessary to determine if the activity of the AAD might be directly or indirectly related to the detoxification process of this monoterpene. Despite that *CaAAD1* is located into a cluster of genes putatively involved in the detoxification process in the genome of *C. americana* (Figure 6), our results of expression show that the other genes associated to this cluster are not expressed (< 5 TPM). However, this result should be taken with care, given that these genes may be expressed under other conditions. Until our knowledge, this gene cluster has not previously been reported in other yeasts or fungi.

On the other hand, our expression findings suggest that genes coding for MDR transporters could be involved in the detoxification process. It is outstanding that the ABC transporter gene (*CaABCG1*) shows the highest transcriptional difference in *C. americana* after being stimulated with (+)-α-pinene. The ABC superfamily has been documented to mediate tolerance to naturally toxic compounds and xenobiotics and/or virulence in many phytopathogenic fungi and yeasts (Coleman and Mylonakis, 2009; Lamping et al., 2010; Coleman et al., 2011; DiGuistini et al., 2011). Based on our phylogenetic analysis the *CaABCG1* transporter of *C. americana* is phylogenetically close to GcABC-G1-3 transporters of *G. clavigera*, a pine pathogen fungus associated with mountain pine beetle *D. ponderosae.* In particular, it has been demonstrated that one of these transporters (GcABC-G1) reduces the intracellular concentration of toxic monoterpenes in this fungus (Wang et al., 2013). The presence and strong expression of this gene under (+)-α-pinene suggest that the CaABC-G1 transporter from *C. americana* might be performing the same function as GcABC-G1 from *G. clavigera*. This might also explain why *C. americana* is one of the dominant members within the gut of *Dendroctonus* species.

Major Facilitator Superfamily proteins have a transport mechanism across biological membranes which depend upon chemo-osmotic gradients for a wide range of substrates including carbohydrates, ions, amino acids, nucleosides, lipids, the expulsion of drugs, and xenobiotic metabolisms of small molecules (Reddy et al., 2012). Studies performed in *C. albicans. C. maltosa, S. cerevisiae, Aspergillus fumigatus*, and *Cryptococcus neoformans* have reported that MFS transporters play an importing role providing resistance to these yeasts to antifungal drugs, low chain organic acids and formaldehyde (Sasnauskas et al., 1992; Tenreiro et al., 2000; Dias and Sá-Correia, 2013; Costa et al., 2014; Redhu et al., 2016). Our results showed that the expression of almost all *MFS* genes are not correlated with the (+)-α-pinene degradation, despite the high expression of some constitutive genes (> 1000 TPM), as well as the overexpression in presence of (+)-α-pinene of the *CaMfs119, CaMfs35*, and *CaMfs86* genes (fold change = 1.80, 1.14, and 1.10, respectively), but which are putative involved with the ion, sugar and amino acid transport, suggesting their participation in physiological processes.

Finally, in the case of MATE transporters, these have been involved in the excretion of metabolic waste products and xenobiotics in prokaryotes and plants, reducing the concentrations of these compounds in cells by vacuolar transport (Moriyama et al., 2008). However, this has been given little attention in yeast, only the Erc1 MATE-type transporter of *S. cerevisiae* has been partially characterized. This protein confers fungal resistance to the methionine analog ethionine (Shiomi et al., 1991). In this study, from the six MATE transporters genes identified and predicted in the vacuole, all presented transcriptional evidence but none was affected by (+)-α-pinene which makes their function still unclear.

In bark beetles, it has observed that α-pinene produce membrane alterations in different cellular organelles (López et al., 2011), and in yeasts, it has been reported that β/α-pinene has also an effect on plasma membranes and mitochondrial functions (Andrews et al., 1980; Uribe et al., 1985). Our findings of GO/KEGG enrichment show that (+)-α-pinene induces genes involved in the lipid metabolic process and the glycerophospholipid metabolism, being these significantly upregulated. At the same time, several genes involved in the transport of membranes are negatively affected; in particular, MFS transporter genes associated to the plasma membrane and other organelles are downregulated (ACS, MCT, SP, SHS, PHS, FHS, and DHA1 families), which suggests that (+)-α-pinene has an effect on the membranes of *C. americana*.

Thus, changes in the lipid membranes, decrease in the membrane permeability and the significant expression of the *CaAAD1* and *CaABCG1* genes could explain the capacity of *C. americana* to overcome the effect of (+)-α-pinene in the gut of bark beetles (e.g., 0.46 ng/gut of (+)-α-pinene). This can maintain the intracellular monoterpenes concentration below its toxic level and mitigate the toxic effect of (+)-α-pinene, allowing to this symbiotic yeast to adapt to living in the gut of *D. rhizophagus* and other *Dendroctonus* species. Further studies focusing on the functions of these proteins are necessary to understand their functional role in the detoxification process in this symbiosis.

In conclusion, *C. americana*, a dominant symbiont of the gut of *Dendroctonus* spp., presented a high variety and abundance of genes families related to the detoxification process, analyses from the transcriptome to the response of the major terpene present in pine trees, α-pinene, showed activity mainly of the Aryl-alcohol dehydrogenase (*CaAAD1*) and the ABC transporter (*CaABCG1*). The evidence suggests that these genes are involved in the capacity of *C. americana* to resist high amounts of monoterpenes in the gut of bark beetles.

## Data Availability Statement

The *Cyberlindnera americana* ChDrAdgY46 whole-genome assembly has been deposited on the National Center for Biotechnology Information (NCBI) under the BioProject number PRJNA531488, BioSample number SAMN11268211, and accession number SSXY00000000. The raw sequence reads of RNA sequencing have been deposited in the NCBI SRA database under the BioProject number PRJNA532616.

## Author Contributions

LS-R, FR-O, and GZ conceived the study. FR-O and GZ acquired funding. LS-R, VT-B, and GZ analyzed and interpreted the data. LS-R and GZ drafted the manuscript. LS-R, VT-B, FR-O, EC-Q, MH-L, and GZ critically revised for important intellectual content and approved the manuscript.

## Conflict of Interest

The authors declare that the research was conducted in the absence of any commercial or financial relationships that could be construed as a potential conflict of interest.

## Footnotes

1 http://www.bioinformatics.babraham.ac.uk/projects/fastqc/

2 http://www.tcdb.org/

3 https://trinotate.github.io/

4 https://transdecoder.github.io/

## Supplementary Material

FIGURE S1 Size distribution of transcripts of *C. americana*.

FIGURE S2 Benchmarking Universal Single-Copy Orthologs (BUSCO) assessment in *Cyberlindnera* species.

TABLE S1 Physicochemical characteristics of gene families associated with the detoxification process.

TABLE S2 Comparison of GO and KOG annotation in the genome and transcriptome of *C. americana.*

TABLE S3 Transcripts per million reads values of genes involved in the detoxification process.

TABLE S4 The 238 differentially expressed genes of *C. americana* under (+)-α-pinene stimulus.

TABLE S5 Results of comparison among the genomes of *C. americana, C. jadinii, C. tropicalis*, and *C. lusitaniae*.

DATA SHEET S1 Gene prediction of *C. americana*.
