# Supplementary figures and images for: An Overview of Genes From Cyberlindnera americana, a Symbiont Yeast Isolated From the Gut of the Bark Beetle Dendroctonus rhizophagus (Curculionidae: Scolytinae), Involved in the Detoxification Process Using Genome and Transcriptome Data
Source: Front Microbiol. 2019 Sep 27;10:2180. doi: 10.3389/fmicb.2019.02180 (PMC6777644; doi:10.3389/fmicb.2019.02180)

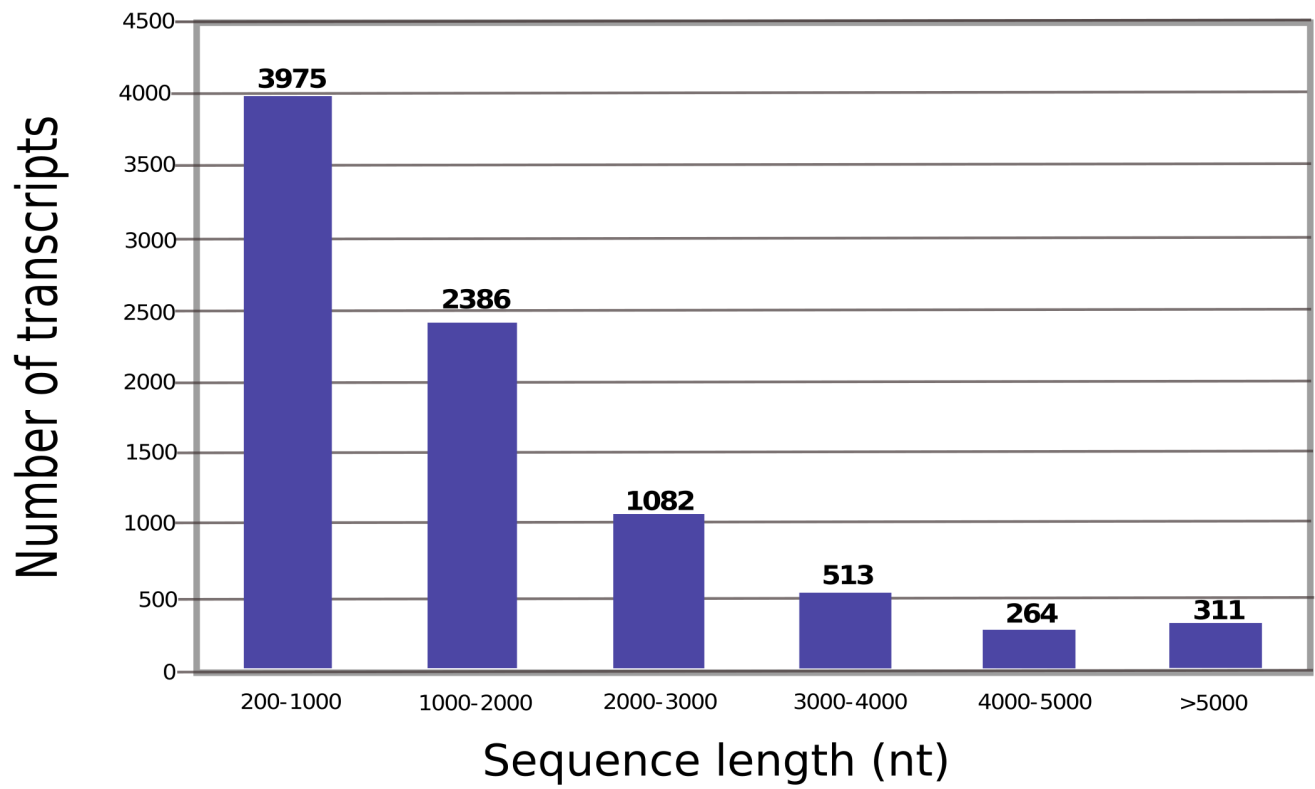

**Figure S1.** Size distribution of transcripts of *C. americana*.

Supplement: FIGURE S1 — Size distribution of transcripts of C. americana. [file Image_1.pdf]

# BUSCO Assessment Results

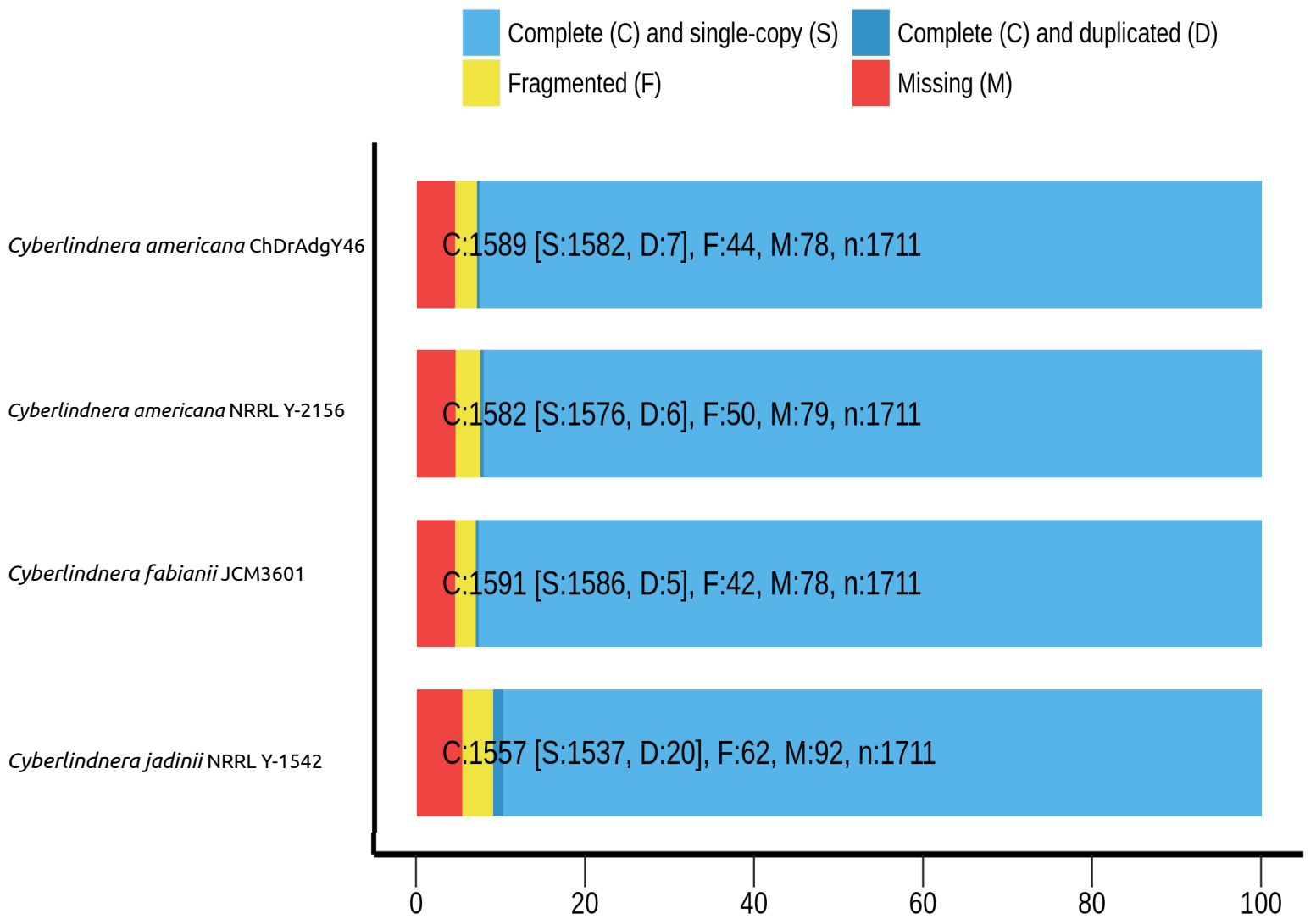

**Figure S2.** BUSCO Assessment in *Cyberlindnera* species

Supplement: FIGURE S2 — Benchmarking Universal Single-Copy Orthologs (BUSCO) assessment in Cyberlindnera species. [file Image_2.pdf]
